# Supplementary material for: Individual Determinants as the Causes of Failure in Learning to Swim with the Example of 10-Year-Old Children
Source: Int J Environ Res Public Health. 2022 May 6;19(9):5663. doi: 10.3390/ijerph19095663 (PMC9100805; doi:10.3390/ijerph19095663)
Supplement: Supplementary file 1 [file ijerph-19-05663-s001.zip › ijerph-1700711-supplementary.pdf]

Supplemantary file S1

Swimming curriculum framework

Lesson No.	Skills	Knowledge
1	Organizational lesson	Familiarization with health and safety rules,<br>Discussing the rules and regulations of the swimming
2	**Preliminary skills test**<br>Water familiarization exercises	
3-5	Getting accustomed to water, adaptation exercises:<br>• breathing exercises,<br>• buoyancy exercises,<br>• spatial orientation under water,<br>• submerging the face, opening the eyes,<br>lying on the chest and the back.<br>Learning of glides. Basic diving	Learning about the characteristics of the aquatic environment.<br><br>Learning the technique of front crawl and backstroke swimming
6	Test I<br>Front glide and back glide	
7-12	Learning lower limb technique for front crawl and backstroke<br>Swimming front crawl and backstroke using crawl movements<br>lower limb movements	Personal hygiene during swimming lessons, correct names, correct use of equipment
13	**Test II**<br>**Backstroke swimming with lower limb crawl**	Good behavior, fair play, and cooperation in a team -classroom, peer support
14-21	Learning of upper limb movements for backstroke swimming:<br>• coordination of upper limb movements with breathing and lower limb movements	Organization of swimming competitions.
22	**Test III**<br>**Backstroke swimming**	Open water safety, use of learned elements in recreational swimming, and encouragement to continue swimming classes (independently or in courses)
23-32	Learning front crawl:<br>• learning upper limb movements,<br>• learning to breathe,<br>• coordination of upper limb movements with breathing and lower limb movements,	Maintaining fitness and good health
33	Test IV<br>Front crawl swimming<br>Preliminaries before competitions	
34	Test for the "I can already swim" badge	
35	Presentation of the "I can already swim" badges<br>Discussion on safe swimming in open waters	

Supplemantary file S1

Assessment of swimming skills

A swimming test was used to assess the swimming skills of the third-grade elementary school students, in which the skills presented were assessed based on direct observation and a scoring system.

In the school year prior to the main research, a pilot study was conducted on third-grade elementary school students to determine the reliability of the tools for assessment of swimming skills and fear of water.

The reliability of the applied swimming tests was evaluated using Spearman's rho correlation analysis with the following variants:

- evaluation of the study by two independent experts at the same time,

- expert evaluation with simultaneous video recording,

- expert evaluation performed twice, one week apart (Arska-Kotlińska et al. 2002).

The assessment of the reliability of the swimming tests involved all three tests, i.e., front glide, backstroke swimming, and front crawl swimming. Children who were able to perform a front glide and swim using backstroke and front crawl were selected for the pilot study. It can be concluded that the tests conducted met the requirements of reliability, as the expert's evaluation was significantly correlated with the evaluation of the other expert, with the video recording, and with the evaluation of the expert for the same subjects on different dates, with very high correlation coefficients (Tab. 1).

Table S1. Evaluation of the reliability of applied swimming skill tests of third-grade elementary school students (Spearman's rho).

Evaluation by two experts				
Skills tests	N	rho	t	p
Front glide	47	0.81	6.91	0.01
Backstroke	47	0.91	14.42	0.01
Front crawl	47	0.88	12.67	0.01
Expert evaluation compared with video recording				

Front glide	47	0.89	8.20	0.01
Backstroke	47	0.90	12.33	0.01
Front crawl	47	0.85	10.95	0.01
Double evaluation by the expert				
Front glide	47	0.82	8.36	0.01
Backstroke	47	0.86	11.59	0.01
Front crawl	47	0.83	13.38	0.01

Skills related to initial adaptation were assessed in the shallow water zone over a distance of up to 7 m, while backstroke or front crawl swimming skills were assessed over a distance of 15 to 25 m. A rule was adopted that the student voluntarily proceeded to other tests, starting with a front glide. After successful completion of the front glide, the subject proceeded to a test assessing backstroke swimming, and after passing this test, they started the front crawl test. Important activities and elements of technique were highlighted for each of the three swimming skills.

Initial adaptation to the aquatic environment: front glide (0-3points):

- submerging the face in the water,
- placing the upper limbs above the head and putting them in the water,
- pulling the lower limbs off the bottom,
- alternating lower limb movements.

Scoring:

0 points - no front glide skills,
1 point - front glide with alternating lower limb movements, submerging the face in the water, placing the upper limbs above the head over a distance of up to 3 m,
2 points - as above, at a distance of 3-5 m,
3 points - as above, at a distance of 5-7 m.

Backstroke swimming (0-6 points):

- head positioning,
- upper body positioning,
- lower limb movements,
- upper limb movements.

Scoring:

0 points - no backstroke swimming skills or swimming a distance of less than 15 m,
2 points - backstroke swimming with alternating lower limb movements and upper

limbs positioned along the upper body,
4 points - backstroke swimming with alternating lower limb movements and non-rhythmic or non-alternating upper limb movements,
6 points - backstroke swimming with alternating lower limb movements and rhythmic upper limb movements.
Front crawl swimming (0-9 points):

- head positioning,
- upper body positioning,
- lower limb movements,
- upper limb movements,
- twisting the head and upper body to breathe in.

Scoring:

0 points - no front crawl swimming skills or swimming a distance of less than 15 m,
3 points - front crawl swimming with alternating lower limb and upper limb movements, without submerging the face in the water and exhaling into the water,
6 points - front crawl swimming with alternating, non-rhythmic lower limb and upper limb movements and exhaling into the water,
9 points - front crawl swimming with alternating rhythmic lower and upper limb movements and exhaling into the water.

The initial swimming test was administered in the first classes but only to the students who gave an affirmative answer when asked by the researcher if they knew how to or had learned to swim. The remaining individuals were considered non-swimmers. Swimming tests during the school year were performed for all children present in swimming classes on a given day. The initial test and subsequent tests consisted, depending on skills, of 1 to 3 trials and took place every 5 lessons, that is, at the 1st and after the 5th, 10th, 15th, 20th, 25th, and 30th lessons.

To ensure the reliability of the assessments used in the swimming tests, direct observation was always conducted simultaneously by two independent experts using new observation sheets each time. The experts did not know the students and had not taught them. Students scored points for the skills they presented.
